# Dried Fruits: Bioactives, Effects on Gut Microbiota, and Possible Health Benefits—An Update

**DOI:** 10.3390/nu15071611

**Published:** 2023-03-26

**Authors:** Cesarettin Alasalvar, Sui Kiat Chang, Penny M. Kris-Etherton, Valerie K. Sullivan, Kristina S. Petersen, Marta Guasch-Ferré, David J. A. Jenkins

**Affiliations:** 1 Life Sciences, TÜBİTAK Marmara Research Center, Gebze 41470, Türkiye; 2 Department of Allied Health Sciences, Faculty of Science, Universiti Tunku Abdul Rahman, Kampar 31900, Malaysia; 3 Department of Nutritional Sciences, Penn State University, State College, PA 16802, USA; 4 Department of Epidemiology, Johns Hopkins Bloomberg School of Public Health, Baltimore, MD 21205, USA; 5 Department of Nutritional Sciences, Texas Tech University, Lubbock, TX 79409, USA; 6 Department of Public Health and Novo Nordisk Foundation Center for Basic Metabolic Research, Faculty of Health and Medical Sciences, University of Copenhagen, 1356 Copenhagen, Denmark; 7 Department of Nutrition, Harvard T.H. Chan School of Public Health, Boston, MA 02115, USA; 8 Departments of Nutritional Sciences and Medicine, Temerty Faculty of Medicine, University of Toronto, Toronto, ON M5S 1A8, Canada; 9 Clinical Nutrition Risk Factor Modification Centre, St. Michael’s Hospital, Toronto, ON M5C 2T2, Canada; 10 Li Ka Shing Knowledge Institute, St. Michael’s Hospital, Toronto, ON M5B 1W8, Canada; 11 Division of Endocrinology and Metabolism, St. Michael’s Hospital, Toronto, ON M5C 2T2, Canada

**Keywords:** dried fruits, gut health and microbiome, cardiometabolic diseases, bone health, dietary guidance

## Abstract

Dried fruits contain many bioactive compounds broadly classified as phytochemicals including phenolics, flavonoids, carotenoids, proanthocyanidins, stilbenes, chalcones/dihydrochalcones, and phytoestrogens. These compounds have antioxidant effects that may benefit health. Dried fruits are also a diverse group of foods with varying fibre contents. The evaluation of the biological activity of these bioactive compounds, including their bioaccessibility and bioavailability, may contribute to the understanding of the health effects of dried fruits. Limited evidence suggests that dried fruits (raisins, cranberries, dates, and prunes) affect human gut microbiota composition in a potentially beneficial manner (in terms of effects on *Bifidobacteria*, *Faecalibacterium prausnitzii*, *Lactobacillus, Ruminococcaceae*, *Klebsiella* spp., and *Prevotella* spp.). There is little epidemiological evidence about the association of dried fruit consumption with cardiovascular disease incidence and mortality, as well as the risk of type 2 diabetes or obesity. Clinical trial evidence for the effects of dried fruit consumption on cardiovascular risk factors, including glycaemic control, is mixed. Clinical trial evidence suggests prunes might preserve bone mineral density in postmenopausal women. Consumption of dried fruits is associated with higher-quality diets. Studies are needed to increase our understanding of the health effects of dried fruits and the underlying biological mechanisms.

## 1. Introduction

Dried fruits are enjoyed by populations worldwide as a shelf-stable, convenient alternative to fresh fruit. Epidemiological evidence suggests dried fruit consumption is associated with lower risk of cardiovascular disease (CVD), type 2 diabetes (T2D), as well as obesity, various cancers, and other chronic diseases, although the evidence is limited and sometimes contradictory. Nonetheless, dried fruits are nutrient dense and a good source of bioactives/phytochemicals [1].

The biological action of bioactives/phytochemicals in dried fruits is dependent on the food matrix release (e.g., bioaccessibility), bioavailability, and metabolism by colonic microbiota [2]. In 2014, the European Food Safety Authority (EFSA) authorized a health claim for dried plums/prunes and gastrointestinal health [3]. This claim states that “*Dried plums/prunes can contribute to normal bowel function*”. To obtain the claimed effect, about 100 g/day (~8–12 prunes, depending on their size) of prunes should be consumed. More recently, the effects of dried fruits and the constituent phytochemicals on microbiota composition and functionality have been active areas of investigation. It is now recognized that microbiota contributes to metabolic health and, when aberrant, the development of cardiometabolic diseases. Thus, identifying dietary strategies to promote metabolic health through microbial modulation is a priority.

Evidence from epidemiological and clinical studies suggests that dried fruit intake may improve glucose metabolism and other cardiovascular risk factors, as well as a lower risk for osteoporosis [4,5]. The intake of dried fruits has also been proposed as a strategy to meet fruit recommendations, improve diet quality, and address nutrient deficiencies [6,7].

This review summarizes evidence on the relationship between dried fruit intake and gastrointestinal (GI) health. Bioactive/phytochemical composition and bioaccessibility and bioavailability of dried fruits are also highlighted. We also discuss the association between dried fruit intake and cardiometabolic diseases, bone health, and diet quality as well as the potential mechanisms involved. This is an emerging area of science, and current evidence suggests that the effects of dried fruits on the microbiome, cardiometabolic disease risk, bone health, and diet quality warrant further investigation.

## 2. Methodologies

To write this narrative review, a detailed literature review was conducted (via sources such as Web of Science, PubMed, SCOPUS, MEDLINE, and Google Scholar). Articles related to the following topics were included: (1) bioactives/phytochemicals present in most commonly consumed dried fruits, (2) bioaccessibility and bioavailability of compounds in dried fruits, (3) GI effects (gut health and microbiota) of dried fruits in animals (in vivo) or human clinical trials, (4) epidemiological evidence about the association of dried fruit consumption with CVD, T2D, and adiposity, (5) cardiometabolic and bone health effects of dried fruit consumption, (6) dietary guidance for dried fruits and benefits on diet quality, and finally, (7) potential mechanisms involved in the observed biological effects. To ensure that current and recent research was presented in this review, only articles published from 2000 onward were included (with a few exceptions due to the relevance of the work), with preference given to articles published between 2015 and 2022 in order to improve contemporary relevance.

Selected articles were examined in detail, and then the bioactives/phytochemicals, bioaccessibility and bioavailability of compounds, gut health and microbiota, epidemiological evidence, cardiometabolic diseases, bone health, and potential mechanisms involved for health benefits as well as diet quality and dietary recommendations for dried fruits were compiled and evaluated. This review is not a systematic review. The most innovative aspect of this review is the update of GI health and cardiometabolic effects of commonly consumed dried fruits (in vivo and in vitro studies). In addition, the update on bioactives/phytochemicals, potential mechanisms involved in the observed biological effects, recommendations for dried fruit consumption, and benefits on diet quality contribute to the novelty of this review.

## 3. Bioactives/Phytochemicals, Dietary Fibre, and Antioxidant Activity in Dried Fruits

Dried fruits contain a variety of bioactive compounds/phytochemicals such as flavonoids (anthocyanins, flavan-3-ols, flavonols, and flavones), proanthocyanidins (dimer, trimer, 4–6 m, and 7–10 m), phenolic acids (hydroxycinnamic acids and hydroxybenzoic acids), carotenoids (α-carotene, β-carotene, β-cryptoxanthin, lutein, and zeaxanthin), and stilbenes as well as phytoestrogens (isoflavones, lignans, and coumestan) and chalcones/dihydrochalcones [1,8,9]. Among these bioactive phytochemicals, phenolic compounds are the major group (Table 1). Alasalvar et al. [9] reported various phenolic compounds (anthocyanins, flavan-3-ols, flavonols, flavones, phenolic acids, proanthocyanidins, chalcones/dihydrochalcones, and stilbenes) in nine dried fruits (apples, apricots, cranberries, dates, figs, peaches, pears, prunes, and raisins). Some dried fruits (such as apricots, cranberries, dates, figs, prunes, and raisins) have the most diverse phenolic profiles. Little information is available about the exact phenolic profiles of dried apples, peaches, and pears. With regard to carotenoids, which are plant pigments responsible for yellow, orange, and bright red hues in many fruits and vegetables, α-carotene, β-carotene, β-cryptoxanthin, and lutein + zeaxanthin are present in dried fruits except raisins (seedless), albeit in varying quantities. Of these, β-carotene is the most abundant in apricots (2163 μg/100 g), peaches (1074 μg/100 g), and prunes (394 μg/100 g) [10]. Phytoestrogens consist of isoflavones, lignans, and coumestans. Apricots, dates, prunes, and raisins have been reported to contain phytoestrogens. Total phytoestrogen content ranged from 30.3 µg/100 g in raisins (seedless) to 445 µg/100 g in apricots. No phytoestrogens have been reported in dried apples, cranberries, figs, and peaches (Table 1) [11]. Detailed quantitative analysis on different classes of phenolic compounds, carotenoids, and phytoestrogens in different forms and varieties of dried fruits are needed.

Dried fruits are a good source of dietary fibre (3.7–9.8 g/100 g) (Table 1). Consumption of dried fruits (around 20–30 g per/day recommended by many countries) provides 10–16% of the recommended daily intake of fibre (14 g/day), depending on the fruit [10,12,13].

The oxygen radical absorbance capacity (ORAC), a measure of antioxidant activity, of dried fruits is relatively high, although it varies by dried fruit type as well as by cultivar/variety (Table 1). For example, raisins (seedless) have the lowest ORAC values (3037 µmol trolox equivalents (TE)/100 g), whereas raisins (Golden seedless) have the highest ORAC value ((10,450 µmol TE)/100 g). Similarly, ORAC values vary appreciably for dates cultivars of Deglet noor and Medjool [14].

**Table 1:** Reported bioactives/phytochemicals, dietary fibre, and antioxidant activity in selected dried fruits.

	Phenolics	Carotenoids (μg/100 g)	Phytoestrogens (μg/100 g)	Dietary Fibre (g/100 g)	Antioxidant Activity (μmol of TE/100 g) ^a^
Apples	Flavan-3-ols Flavonols Phenolic acids Chalcones/dihydrochalcones	Lutein + zeaxanthin (18)	-	8.7	6681
Apricots	Flavan-3-ols Flavonols Flavones Phenolic acids Chalcones/dihydrochalcones	β-Carotene (2163)	Isoflavones (39.8) Lignans (401) Coumestan (4.2)	7.3	3234
Cranberries	Anthocyanins Flavan-3-ols Flavonols Phenolic acids Proanthocyanidins	β-Carotene (27) Lutein + zeaxanthin (138)	-	5.3	-
Dates	Anthocyanins Flavonols Phenolic acids Proanthocyanidins	β-Carotene (6) Lutein + zeaxanthin (75)	Isoflavones (5.1) Lignans (324) Coumestan (0.8)	8.0	2387–3895 ^b^
Figs	Anthocyanins Flavan-3-ols Flavonols Flavones Phenolic acids Proanthocyanidins	β-Carotene (6) Lutein + zeaxanthin (32)	-	9.8	3383
Peaches	Anthocyanins Flavan-3-ols Flavonols Phenolic acids	α-Carotene (3) β-Carotene (1074) β-Cryptoxanthin (444) Lutein + zeaxanthin (559)	-	8.2	4222
Pears	Flavan-3-ols Phenolic acids Chalcones/dihydrochalcones	β-Carotene (2) Lutein + zeaxanthin (50)	-	7.5	9496
Prunes	Flavan-3-ols Flavonols Phenolic acids	α-Carotene (57) β-Carotene (394) β-Cryptoxanthin (93) Lutein + zeaxanthin (148)	Isoflavones (4.2) Lignans (178) Coumestan (1.8)	7.1	8578
Raisins	Anthocyanins Flavan-3-ols Flavonols Flavones Phenolic acids Stilbenes	-	Isoflavones (8.1) Lignans (22) Coumestan (0.2)	3.7	3037–10,450 ^c^
References	[1,9]	[10]	[11]	[10]	[14]

^a^ Based on oxygen radical absorbance capacity (ORAC). ^b^ Between Deglet noor and Medjool cultivars. ^c^ Among white, seedless, and Golden seedless raisins.

Several studies have reported the bioactive compounds and antioxidant activities of dried fruits are higher than those of their corresponding fresh counterparts [15,16,17,18]. This is due to bioactive compounds and antioxidants becoming concentrated after the drying process. However, losses (e.g., carotenoids and anthocyanins) or changes in some compounds occur during drying and storage. Therefore, drying types and duration, as well as storage and packaging are of great importance in terms of functional/nutritional quality and flavour (taste and aroma) of the final product for consumption.

## 4. Bioaccessibility and Bioavailability of Compounds in Dried Fruits

The bioaccessibility and bioavailability of compounds in dried fruits have been investigated using in vitro models. These models mimic human in vitro GI digestion (e.g., mouth (oral or salivary digestion), stomach (gastric digestion), small intestine (intestinal digestion), and colon or large intestine (colonic digestion)) [19,20,21,22]. Bioaccessibility refers to the level of a compound released from the food matrix during GI digestion that becomes available for absorption (bioavailability) in the intestine [23]. To exert health effects, ingested compounds, including phytochemicals and micronutrients (vitamins and minerals) contained in food, must be released from the food matrix in the GI tract and become bioavailable [21].

Evidence suggests that the phenolics contained within dried fruits are bioaccessible. Recently, Scrob et al. [24] investigated the bioaccessibility of constituents in six dried fruits (dates, raisins, coconuts, cranberries, prunes, and bananas) and demonstrated the highest bioaccessibility of phenolics was observed in prunes and the lowest in cranberries and dates. Total sugars content increased after in vitro digestion of coconuts, dates, and raisins, but it decreased for bananas, cranberries, and prunes. In vitro digestion led to an increase in the antioxidant activity for most dried fruits. This study showed prunes, coconuts, bananas, and raisins are sources of high bioaccessible phenolics. However, the contribution of dried fruit consumption to the recommended dietary allowances (%) was less considering the bioaccessible fraction compared to the total content.

Polar phenol bioaccessibility of dates using a static model of in vitro digestion was also investigated by Panagopoulou et al. [22]. Simulated GI digestion revealed date polar phenols were found to be bioaccessible to an extent depending on the polar phenol class, the nature of the polar phenols, and the specific date matrix. A 37–70% release was observed post-oral digestion, in terms of total phenolic content, which further increased post-gastric digestion (>100%).

Ma et al. [20] investigated the biological activities of kiwifruits and kiwifruit products including dried slices under simulated GI in vitro digestion. Dried slices showed the lowest biological activity compared to those of other kiwifruit products (such as raw fruit, juice, vinegar, wine, yogurt, and jelly). However, dried slices and jam had the highest quantity of minerals (per unit weight). Thus, consuming dried slices and jam could supply more mineral elements than other forms of the fruit [20].

The impact of GI digestion on the total phenolic content (TPC) and antioxidant activities of dried apricots, figs, and raisins was evaluated by Kamiloglu et al. [25]. There was an increase in TPC (0.4–4.5-fold) for all samples after the gastric digestion. The antioxidant activities of dried apricots and figs were increased as determined by various antioxidant activity assays.

In conclusion, in vitro GI digestion studies have some advantages including being fast and inexpensive, without human ethics concerns. However, these digestion systems (static and dynamic) might not completely mimic human physiology. In vitro models need to be compared with in vivo models (particularly, human intervention studies) to better understand the biological effects. These comparative data are essential for demonstrating the biological relevance of bioactive compounds in the context of nutrition and human health [1,25].

## 5. Dried Fruits, Gut Health, and Microbiota

Diet is an important modulator of the gut microbiota and its metabolite production. The multiple interactions between food components and gut microbiota as well as the modification of the gut microbiota composition and activities by food components contribute to human health [26,27]. To the best of our knowledge, few studies have investigated the effects of dried fruit intake on gut microbiota. Recent findings from animal and human studies are reviewed.

### 5.1. In Vivo Animal Studies

A recent chapter by Muñoz and Lamuela-Raventós [28] reviewed the effects of different dried fruits on gut health and microbiota composition using in vitro and in vivo studies. These in vivo studies were conducted using rat, fish, or broiler chick models. The effects of dried fruits on the modulation of gut microbiota from preclinical studies published following the chapter by Muñoz and Lamuela-Raventós [28] are discussed in this section. Among dried fruits, gut health and the microbiota data are available for goji berries, prunes, and dried cranberries.

In a recent study conducted by Cremonesi et al. [29], New Zealand white rabbits fed chow with 3% goji berries had enrichment of *Ruminococcaceae*, *Lachnospiraceae*, *Lactobacillaceae*, and the genus *Lactobacillus*, all of which are considered to be beneficial bacteria, compared to a control group fed regular chow. In addition, the supplementation of goji berries enhanced lactic acid fermentation that contributes to the caecal fermentation [29]. Similarly, in a 10-week study involving mice, Tian et al. [30] demonstrated that supplementation of goji berries at 1.5 or 3% modulated the gut microbiota composition by enhancing the growth of beneficial bacteria such as *Verrucomicrobia*, *Bacteroidetes*, *Bacteroidales S24-7* group, *Anaerotruncus*, *Coprococcus 1*, *Ruminococcaceae UCG-014*, and *Akkermansia*, while suppressing the growth of harmful bacteria such as *Firmicutes*, *Helicobacter*, *Bacteroides*, and *Mucispirillum*. Meanwhile, administration of goji berries promoted the growth of short-chain fatty acid (SCFA)-producing bacteria, increasing the production of SCFAs [30]. Finally, Kang et al. [31] studied interleukin (IL)-10-deficient mice and showed feeding with goji berries (1% of dry feed weight) for 14 days enhanced the abundance of *Bifidobacteria* and butyrate-producing bacteria, *Clostridium leptum*, and its dominant constituent *Fecalibacterium prazusnitzii,* compared to control chow fed mice. This resulted in an increase in faecal butyrate content [31].

In another study, the effects of freeze-dried cranberries in dextran sodium sulphate-treated (DSST) male CD-1 mice (to induce colitis) were evaluated [32]. This study showed supplementation with 1.5% (*w*/*w*) freeze-dried cranberries (equivalent to 7.5 g of whole cranberry powder) alleviated colitis in DSST mice by reducing the levels of numerous pro-inflammatory cytokines. In addition, treatment with freeze-dried cranberries alleviated the reduced α-diversity of the gut microbiota induced by DSST [32]. Specifically, treatment with freeze-dried cranberries enhanced the abundance of beneficial bacteria, such as *Bifidobacterium* and *Lactobacillus* while reducing the abundance of harmful bacteria, such as *Suterella* and *Bilophila* [32].

### 5.2. Human Clinical Trials

Wijayabahu et al. [33] conducted a human clinical trial evaluating the effect of three servings (28.3 g per serving) of sun-dried raisins daily for 14 days on gut microbiota composition in healthy adults. Overall gut microbiota composition was not different after raisin consumption, but specific operational taxonomic units (OTUs) were affected. For example, OTUs matching *Faecalibacterium prausnitzii* and *Ruminococcaceae* were significantly enhanced, while OTUs matching *Klebsiella* spp. and *Prevotella* spp. were reduced significantly. These taxa, *Faecalibacterium prausnitzii* and *Ruminococcaceae*, are important for the breakdown of complex carbohydrates in the gut microbiota [33]. Meanwhile, the reduction in OTUs matching *Klebsiella sp.* and *Prevotella sp.* indicated a reduced risk for urinary tract infections and chronic inflammation, respectively [34].

In another randomized, double-blind, cross-over, controlled trial, healthy adults consumed 30 g/day of freeze-dried whole cranberry powder or a placebo for 5 days [35]. Cranberry powder consumption decreased the abundance of *Firmicutes*, while increasing the abundance of *Bacteroidetes*. In addition, the consumption of freeze-dried cranberry powder reduced the production of secondary bile acids and prevented the reduction in SCFAs, relative to the control [35]. Bekiares et al. [36] demonstrated that intake of 42 g/d of sweetened dried cranberries (SDC) for 14 days increased the *Firmicutes*:*Bacteroidetes* ratio and the relative abundance of *Akkermansia*. The authors recommended that further studies be conducted using well-controlled study designs and larger sample sizes to better understand the effect of cranberries on the relative abundance of *Akkermansia* [36].

The effect of prunes on bowel function has also been investigated [37]. Healthy adults (*n* = 120) consumed either 80 g or 120 g of prunes daily for 4 weeks, with stool weight and frequency as the primary study outcomes. Participants who consumed both 80 g and 120 g of prunes daily had higher stool weight and frequency than the control group. Supplementation with prunes significantly enhanced the relative abundance of *Bifidobacteria* compared to the control group. However, supplementation of prunes did not affect the levels of SCFA or stool pH in the subjects studied. The authors postulated that the effect of prunes on the gut microbiota could be mediated by fibre content, sorbitol, or phytochemicals in prunes [37]. Hence, more research should be carried out to confirm this result. A recent randomized, open-label, controlled trial evaluated the effects of prune consumption in adult women after undergoing benign gynaecologic surgery [38]. Participants (*n* = 77) consumed 12 prunes with 100 g docusate sodium (widely used as medicine as laxative and as stool softener) twice daily vs. docusate alone for 3 days. Participants who consumed 12 prunes twice daily had an increased likelihood of a bowel movement and earlier hospital discharge than the control group [38].

Dates have also been tested in a randomized, controlled, cross-over, clinical trial for gut microbiota, and GI function [39]. Healthy adult participants consumed 50 g of dates per day or maltodextrin-dextrose as a control for 21 days, with a 14-day washout period. Adults who consumed prunes daily had higher stool weight and bowel movement frequency than the control group. Supplementation with dates did not cause any significant alterations in the SCFA levels or in the growth of selected bacteria [39].

**Figure 1:**
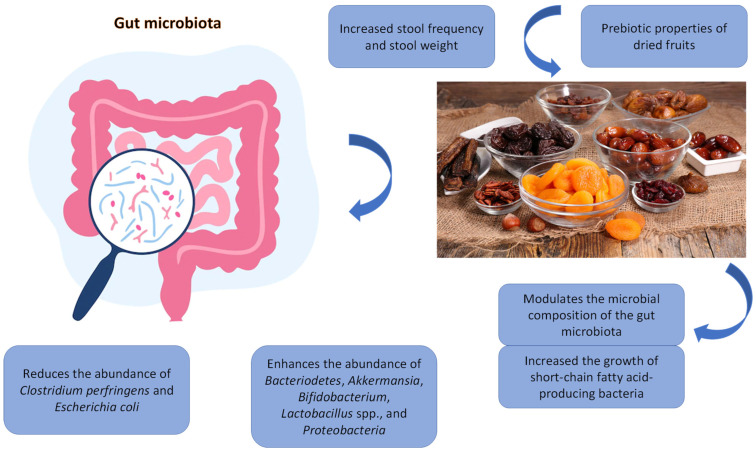
Potential mechanisms of action of dried fruit-related gut microbiota modulation.

Most studies conducted to date have examined the effects of dried fruits on microbiota composition, whereas studies on metabolite production and functionality are scarce. Figure 1 summarises the potential mechanisms by which intake of dried fruits may modulate gut microbiota to influence health. Phytochemicals from dried fruits undergo significant biotransformation by gut microbiota, and the resulting metabolites may influence health [40]. Future studies, including in vitro, animal, human, and mechanistic studies are needed to address this research gap.

## 6. Epidemiological Evidence for Health Benefits of Dried Fruits

### 6.1. CVD

CVDs are the leading cause of death worldwide [41]. A suboptimal diet is a major contributor to cardiovascular mortality, with low fruit intake ranked among the top three global dietary risk factors for cardiovascular deaths [42]. Individuals consuming dried fruits within the context of healthy dietary patterns generally have a healthier cardiometabolic risk profile, with lower lipid concentrations, blood glucose, and blood pressure [43,44,45]. However, there is limited evidence regarding the impact of dried fruit consumption on cardiovascular risk factors, CVD incidence, and mortality. Intake of grapes and raisins (queried together) ≥4 servings/week was associated with an 8% lower risk of hypertension in the Nurses’ Health Study and Health Professionals Follow-Up Study cohorts, which included 187,453 individuals. Dried plum intake was not associated with incident hypertension after adjusting for other cardiovascular risk factors (including body mass index—BMI) and lifestyle and dietary factors [46]. Dried fruit consumption (≥1 vs. <1 serving/day) was not associated with cardiovascular mortality in the Massachusetts Health Care Panel Study, though few people (~5%) consumed more than 1 serving of dried fruit daily [47]. In the UK Women’s Cohort Study, combined fresh and dried fruit intake was associated with a lower risk of CVD mortality (8% lower risk per 80 g/day) [48]. However, just dried fruit intake was not significantly associated with cardiovascular mortality.

### 6.2. T2D

In the Nurses’ Health Study and Health Professionals Follow-Up Study cohorts after adjusting for demographic, lifestyle, dietary factors, and diabetes-related risk factors including BMI, every three servings/week of grapes and raisins was associated with a 12% lower risk of T2D [49]. Greater dried plum intake was not associated with T2D incidence. Substituting equivalent portions (three servings/day) of dried plums or grapes and raisins for fruit juice was associated with an 18–19% lower diabetes risk [49]; however, the association may be attributable to fibre intake rather than dried fruit intake per se.

### 6.3. Body Weight

Observational evidence suggests that dried fruit intake is associated with a lower risk of excess adiposity. Based on the most recent cross-sectional analysis of data from the National Health and Nutrition Examination Survey (NHANES; from 2007 to 2016), dried fruit consumers had a lower mean BMI (−0.8, 99% CI −1.4 to −0.2; *p* = 0.002) and waist circumference (−2.6 cm, 99% CI −4.2 to −0.9 cm; *p* < 0.001) than non-consumers [50]. Mean dried fruit intake in US adults was 0.04 ± 0.001 cup-equivalents/day, which represented 3.7% of total daily fruit intake. In an earlier NHANES analysis (from 1999 to 2004), dried fruit consumers (≥⅛ cup-equivalent per day) had lower body weight (78.2 ± 0.6 vs. 80.7 ± 0.3 kg; *p* < 0.01) and BMI (27.1 ± 0.2 vs. 28.1 ± 0.2 kg/m^2^; *p* < 0.01) than non-consumers [51]. In another NHANES analysis (from 2001 to 2012), raisin consumption (defined as having any amount during the first 24 h dietary recall) was associated with a lower body weight (−4.2%), BMI (−5.2%), and waist circumference (−3.8%) [52]. Raisin consumers were 39% less likely to have overweight or obesity.

In summary, few epidemiological studies report favourable associations between dried fruit and CVD, T2D, and body weight, but health benefits are not consistently shown. Observed associations between dried fruit intake and CVD, T2D, and body weight may be confounded by overall diet quality as well as other health-promoting behaviours. While many studies adjust for some foods and nutrients, the specific dietary components included in models vary widely. More comprehensive adjustment for dietary and lifestyle factors may strengthen future epidemiological studies investigating dried fruit consumption. In addition, the ability to detect associations between dried fruit intake and health may be limited by low observed consumption in these study populations. Investigating health associations in populations that routinely consume greater amounts of dried fruits could yield stronger evidence.

## 7. Clinical Trial Evidence for Dried Fruits and Health

### 7.1. Cardiometabolic Diseases

In several clinical studies, dried fruit intake has improved cardiovascular risk factors, including cholesterol, blood pressure, and glycaemic control (Table 2). However, the effects are inconsistent, which may be attributable to differences in the bioactive phytochemical and nutrient profiles of dried fruits, as well as differences in trial designs.

Two studies reported improved low-density lipoprotein cholesterol (LDL-C) after prune consumption compared to energy-matched control foods. In a randomized crossover trial, LDL-C was 0.17 mmol/L (~6.6 mg/dL) lower after 41 hypercholesterolemic men consumed 12 (~100 g) prunes vs. a 360 mL portion of grape juice daily for 4 weeks [53]. In a parallel design trial, adults with overweight and obesity randomized to consume two daily 100-calorie prune snacks (~84 g/day) for 8 weeks had lower LDL-C (−24.5 mg/dL) compared to the control arm randomized to consume low-fat muffins [54]. In two other parallel design studies, cholesterol reductions were observed in participants who consumed dried fruits, but the changes did not differ relative to the control group. Consumption of three dates/day for 16 weeks reduced total cholesterol (−0.209 mmol/L) in adults with T2D, while changes in LDL-C and high-density lipoprotein cholesterol (HDL-C) were not significant [55]. Among adults with hyperlipidaemia, consuming 90 g/day of raisins for 5 weeks reduced total cholesterol (−0.72 mmol/L) and LDL-C (−0.68 mmol/L) [56]. In all four of these studies, diet records confirmed that participants maintained constant energy intakes across the study periods that were consistent with baseline intakes, and three of the four studies confirmed that weight remained stable throughout the study duration [54,55,56].

In contrast, several studies have shown either no change or increased cholesterol after dried fruit consumption. Three studies reported no effect of raisins on total or LDL-C compared to the usual diet [57] or energy-matched processed snacks [58,59]. In a randomized crossover trial in adults with above optimal or high LDL-C (100–189 mg/dL; *n* = 102), daily consumption of 120 g of dried Mission figs for 5 weeks increased total cholesterol compared to usual diet, though neither LDL-C nor HDL-C significantly differed between conditions [60]. Energy intake was approximately 200 kcal greater on the fig condition, resulting in a small statistically non-significant 0.4 kg weight gain. Among adults with overweight and obesity, average total and LDL-C did not differ after 4 weeks of daily consumption of ¾ cup of mixed dried fruits (comprising equal parts raisins, dates, prunes, and dried figs) compared to calorie- and carbohydrate-matched processed snacks, though LDL-C increased 0.10 mmol/L (~4 mg/dL) from baseline after dried fruit consumption [45]. While no diet records were collected, small (0.3–0.4 kg) weight gains were observed after both conditions, suggesting that study foods were not completely substituted for other dietary energy sources. Differences in energy balance may be an important explanatory factor distinguishing trials that demonstrate the cholesterol-lowering effects of dried fruits vs. those that do not.

Several studies have demonstrated blood pressure-lowering effects of raisins, but not other dried fruits. Both systolic and diastolic blood pressure (SBP and DBP) were reduced in adults with overweight or obesity who consumed three 1-ounce portions of raisins (84 g) daily for 12 weeks, compared to energy-matched processed snack foods [58]. SBP decreased with raisin consumption in a similarly designed study among adults with T2D [59], while a 36 g portion of Corinthian raisins consumed daily for 24 weeks improved DBP in adults with T2D, compared to the control arm consuming their usual diets [57]. Daily consumption of 90 g of raisins for 5 weeks also reduced DBP in adults with hyperlipidaemia, compared to the usual diet control arm [56]. In contrast, ¾ cup of mixed dried fruits did not improve resting brachial, 24 h ambulatory, or central blood pressure compared to processed snacks in adults with overweight or obesity [45]. Prune consumption (84 g/day for 8 weeks) also did not reduce blood pressure compared to processed snacks [54].

The effect of dried fruit intake on glycaemic control is important given that they are high in natural sugars. Acutely, dried fruits have a low-to-moderate glycaemic index and can attenuate glycaemic response when substituted for refined carbohydrates [61,62], likely due to partial displacement of glucose with fructose. A lower glycaemic index diet has been associated with a lower risk of CVD and mortality [63].

Several studies have shown that routine dried fruit consumption does not adversely affect glycaemic control. In adults with overweight and obesity, a daily intake of 3 ounces of raisins for 12 weeks reduced haemoglobin A1c (HbA1c) (−0.08%) compared to energy-matched processed snacks, while fasting glucose was unchanged [58]. Among adults with T2D, daily consumption of 3 ounces/day of raisins for 12 weeks did not alter HbA1c or fasting glucose compared to calorie-matched snacks [59]. Similarly, compared to the usual diet, daily consumption of dates (3 g/day for 16 weeks) [55] or Corinthian raisins (36 g/day for 24 weeks) [57] did not affect HbA1c in adults with T2D.

In contrast, two studies showed adverse effects of dried fruits on glycaemic control. Daily prune intake (84 g) for 8 weeks increased C-peptide, which is released during insulin production [54]. However, fasting glucose and insulin concentrations were not altered, and the increase in C-peptide did not significantly differ from the control arm. In adults with overweight and obesity, a small increase in fasting glucose (0.08 mmol/L, ~1.4 mg/dL) was observed after 4 weeks of consuming ¾ cup/day of mixed dried fruits compared to calorie- and carbohydrate-matched processed snacks [45]. Since glycaemic measures were not the primary focus of the trial, these findings should be interpreted with caution and require replication.

Overall, the evidence is mixed regarding the effect of dried fruit consumption on cardiovascular risk factors. While several clinical studies show reductions in cholesterol and blood pressure, without harm to glycaemic control, benefits are not consistently observed. Additional well-designed randomized controlled trials that account for the potential confounding effect of changes in energy intake and body weight are needed to confirm the cardiovascular benefits of dried fruit consumption.

### 7.2. Bone Health

Preclinical studies conducted in rodent models of osteopenia or osteoporosis show prune supplementation prevents and reverses bone loss by modulating oxidative and inflammatory pathways [64,65,66]. Inflammation and oxidative stress enhance bone resorption by increasing osteoclast function and suppress bone formation through reducing osteoblast function [64]. These preclinical findings are generally supported by evidence from clinical trials. Other dried fruits have not been linked improved bone health.

Five clinical trials have been conducted in postmenopausal women and provide suggestive evidence that intake of prunes (50 or 100 g/day) for 3 to 12 months may have osteoprotective effects [67,68,69,70,71]. Four trials conducted by one research group show potential antioxidant and anti-inflammatory effects [67,68,69], as well as improvements in markers of bone formation [70] and resorption [67,68]. Hooshmand et al. [67] showed 100 g/day of prunes increased bone mineral density (BMD) of the ulna and spine compared to 75 g of dried apple after 12 months; no change in neck of femur, total hip, or total body BMD was observed. In a subsequent study, this group demonstrated 50 and 100 g/day of prunes attenuated loss of total BMD compared to the control group after 6 months; no effects were observed for total hip, L_1_–L_4_ lumbar vertebra, or ulna BMD [68]. In a recent, single-centre, parallel-arm 12-month randomized controlled trial, 50 g/day of prunes preserved total hip BMD (–0.3 ± 0.2%) compared to the control group (–1.1 ± 0.2%) in postmenopausal women. An intake of 100 g/day of prunes did not affect BMD; however, the dropout rate was 41% for this group, suggesting limited feasibility of this dose [71]. This large, well-conducted randomized controlled trial is generally confirmatory of previous trials, and the totality of the evidence suggests intake of 50 g/day of prunes might be an efficacious, non-pharmacological intervention to preserve BMD in post-menopausal women.

Recently, two small studies examining the effect of prune intake on markers of bone metabolism in older men have been conducted [72,73]. In a 3-month randomized controlled trial of men 55–80 years with mild bone loss, 50 g/day and 100 g/day of prunes had limited and inconsistent effects on markers of bone turnover compared to the control group [72]. In a subsequent 12-month study by the same group, 100 g/day of prunes did not affect total body, spine (L1–L4), hip, and ulna BMD [73]. The findings of these studies should be cautiously interpreted given the small sample sizes examined limiting statistical power.

## 8. Dried Fruits and Diet Quality

**Figure 2:**
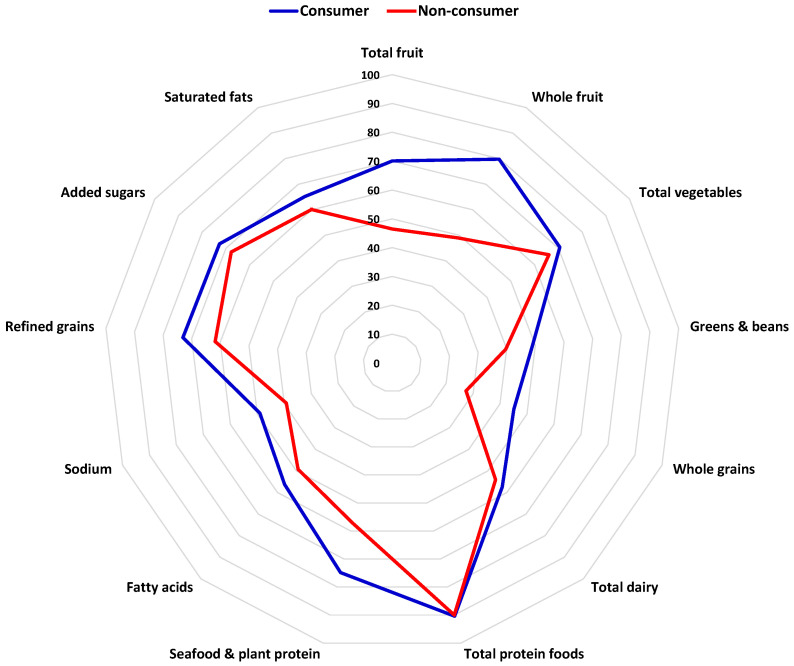
HEI-2015 component scores for dried fruit consumers and non-consumers, NHANES 2007–2016. Dried fruit consumers reported ≥ ¼ cup-equivalent dried fruit intake on at least one of two 24 h diet recalls. Component scores are represented as percentages of maximum score. Data from [50]. Copyright Elsevier (2021).

Individuals who consume dried fruits tend to have higher-quality diets, overall. Based on an analysis of NHANES data from 2007–2016, adult consumers of dried fruits had higher Healthy Eating Index (HEI)-2015 scores, representing better adherence to the 2015–2020 Dietary Guidelines for Americans [50]. Specifically, they had higher intakes of fruits, vegetables, whole grains, legumes, seafood, and plant proteins, and they had lower intakes of (and thus higher HEI-2015 component scores for) sodium, refined grains, and saturated fats (Figure 2) [50]. Consumed as snacks or incorporated into meals, dried fruits can contribute to a healthy dietary pattern. In US adults, dried fruit consumption adds to total fruit intake, rather than displacing other forms of fruit, and contributes to greater intakes of dietary fibre and potassium [50]. Thus, increasing dried fruit intake could be an effective strategy to increase intakes of fruit, fibre, and potassium.

## 9. Dietary Recommendations for Dried Fruit Consumption

A suboptimal diet is a leading cause of morbidity and mortality globally, and a suboptimal fruit intake is a major contributor to CVD, diabetes, and neoplasms [42]. A healthy dietary pattern that includes fruits is the basis for current dietary recommendations made by many organizations globally. In 2020, the World Health Organization recommended a healthy diet that includes the following: at least 400 g (e.g., five portions) of fruit and vegetables per day, excluding potatoes, sweet potatoes, cassava, and other starchy roots [74]. The 2020–2025 US Dietary Guidelines for Americans recommends two cup equivalents of fruits per day (per 2000 calories), which is equal to four servings per day [6]. With respect to dried fruits, ¼ cup is equal to a ½ cup serving of fruit. The European Commission (the European Union as well as Iceland, Norway, Switzerland, and the UK) recommends two to three servings per day of fruit [7]. According to the Global Burden of Disease Study 2017, fruit consumption (94 g per day) falls short of current (two to three servings per day) dietary recommendations for fruit [42]. Similarly to the US, some European countries include dried fruit in the fruit recommendations, whereas others have specific recommendations for dried fruit in the range of 20–30 g per day [7]. According to 2020–2021 data from International Nuts Council, annual global per capita dried fruit consumption is about 1.2 g per day [75].

## 10. Potential Mechanisms Involved for Health Benefits of Dried Fruits

Among dried fruits, prunes are an excellent source of vitamin K, providing about 28 μg of vitamin K per serving of five prunes (47.5 g), which is 23% of the recommended dietary allowances for men and 31% for women [2,10]. Although no bioavailability studies have been conducted for vitamin K in prunes, vitamin K absorption is significantly increased in the presence of some dietary fat. Thus, consuming prunes in the absence of fat, for example, alone as a snack, may result in relatively low absorption of their vitamin K [2]. In general, vitamin K plays a role in blood clotting, bone metabolism, and regulating blood calcium levels. Dried fruits are a good source of potassium [10]. It is likely that potassium is well absorbed from dried fruits and therefore would be a significant dietary source of the mineral. It has been reported that a high sodium:potassium ratio is associated with a significant increase in the risk of CVD and all-cause mortality in a US population [76]. Increased consumption of dried fruits would provide a means to reduce the sodium-potassium ratio, therefore potentially reducing CVD risk.

Dried fruits are rich in bioactives/phytochemicals, such as phenolic compounds, phytoestrogens, and carotenoids with potent antioxidant capacities [1,8,9]. These compounds scavenge free radicals and hence alleviate the oxidative stress that causes tissue damage, aging, and other chronic diseases [9]. Studies using simulated in vitro GI track digestion models indicate that there is little absorption of bioactives/phytochemicals from dried fruits after intestinal digestion. It has been hypothesized that unabsorbed bioactives/phytochemicals might be active in the digestive tract, rather than systemically. Because the digestive tract is a major organ involved in the immune response, effects within it may still contribute significantly to its overall health indirectly. In the colon, bioactives/phytochemicals are metabolized by the gut microbiota to form a wide range of metabolites, some of which would be responsible for health benefits attributed to the parent compounds. Therefore, measuring nutrient bioavailability from dried fruits is an open area of investigation [2]. Furthermore, the consumption of dried fruits modulates the diversity of gut microbiota by enhancing the relative abundance of beneficial microbes while reducing the relative abundance of harmful microbes [31,32]. Modulation of the gut microbiota by dried fruit consumption alleviated chronic inflammation, which in turn reduces the severity of metabolic disorders, such as CVD, T2D, and obesity [34].

In vitro and in vivo studies have been conducted to elucidate the mechanisms by which dried fruits may promote improvement in glycaemic control and insulin sensitivity. In human studies, dried fruits have a low-to-moderate glycaemic index. Mechanisms that may help to explain the benefits of dried fruits may relate to their relatively lower glycaemic index and insulin index potential, high mineral content of potassium and magnesium, and increased fibre content, as well as high levels of antioxidant and bioactives/phytochemicals [77].

Obesity is a disease characterized by chronic accumulation of excessive fat in adipose tissues, which leads to the production of pro-inflammatory cytokines. Chronic inflammation causes endothelial dysfunction, accompanied by insulin resistance. In addition, obesity, high blood pressure, high glucose levels, abnormal lipids/lipoproteins [total cholesterol, LDL-C, and triacylglycerols (TAG)], and chronic inflammation are major risk factors for atherosclerosis. Inhibition of inflammatory pathways by numerous bioactives/phytochemicals contained in dried fruits may beneficially affect inflammation-related diseases (e.g., metabolic disorders, such as CVD and T2D) [45,54].

Evidence suggests that prunes both prevent and reverse bone loss in postmenopausal women and potentially in men. Dried fruits, in general, contain several bioactives/phytochemicals (including but not limited to resveratrol, kaemferol, proanthocyanidins, quercetin, cholorogenic acid, and catechin) with potential osteoprotective effects; however, the mechanisms by which these effects occur remain unclear [5].

In short, the potential of dried fruit to be a therapeutic strategy to prevent the severity of numerous chronic metabolic diseases warrants further investigation.

**Figure 3:**
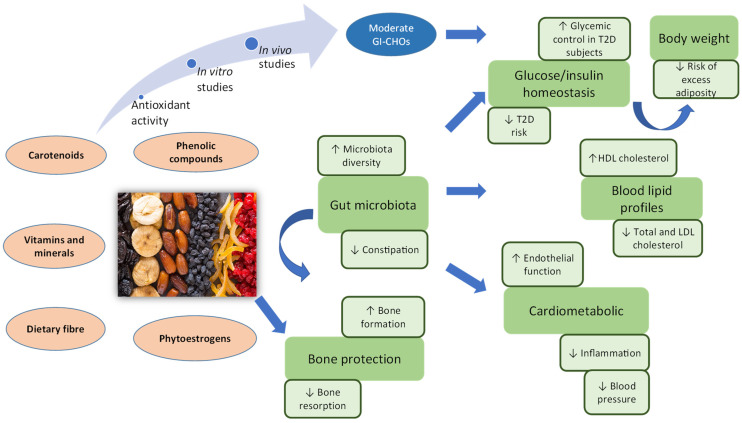
Summary of the health benefits ascribed to dried fruits. Frequent consumption of dried fruits benefits cardiovascular, gut microbiota, and bone health due to their unique composition of nutrients, bioactives/phytochemicals, and fibre. Abbreviations: CHOs, carbohydrates; HDL, high-density lipoprotein; GI, glycaemic index; LDL, low-density lipoprotein; T2D, type 2 diabetes.

The relationships between bioactive compounds/phytochemicals present in dried fruits and health outcomes are summarized in Figure 3.

## 11. Limitation of Studies in Dried Fruits and Future Recommendations

The bioactives/phytochemicals, gut microbiota, and bioavailability as well as health benefits of dried fruits have been less explored compared to their fresh counterparts. Although the bioactive/phytochemical profiles of some dried fruits (such as apricots, cranberries, dates, figs, prunes, and raisins) are well known, limited evidence is available for dried berries and tropical/non-tropical dried fruits. In addition, information about the bioavailability of minerals, vitamins, and bioactives/phytochemicals from dried fruits is scarce [1,8,9]. Measuring bioavailability of nutrients and bioactives/phytochemicals is an active area of research. More research needs to be conducted to determine circulating metabolite profiles after ingestion of dried fruits compared to fresh fruit counterparts [2]. Few studies have investigated the effect of dried fruits on gut microbiota, and further research is needed to understand the health implications of dried fruit related gut microbiota modulation [28]. Further research is also needed to clarify the extent to which bioactives/phytochemicals are altered by processing and whether this affects their bioactivity. Elucidating the mechanisms and bioactives/phytochemicals responsible can also help to identify processing techniques (such as sun-drying vs. heat-drying vs. freeze-drying) or particular fruits that best promote cardiometabolic and bone health [78]. Finally, evidence suggests that prunes have beneficial effects on bone health and may prevent osteoporosis. Further research is needed to examine the effectiveness of this potential non-pharmacological intervention given the side effects of pharmacological therapy for osteopenia [5].

## 12. Conclusions

Research about the health benefits (e.g., specifically related to the microbiota, cardiometabolic diseases, and bone health) of dried fruits is in its early stages. The phytochemical profiles of different dried fruits have been investigated; however, our understanding of their bioaccessibility and bioavailability is not well understood. Furthermore, a better understanding of the biological effects of dried fruits and their bioactive compounds on cardiometabolic diseases, their risk factors, bone health, and the microbiome is needed. Despite this, we have an understanding that is evolving about the health benefits of some of the bioactive compounds in dried fruits and also the many health benefits of fresh fruits and juices. The encouraging results from the studies with both dried fruits, as well as fresh fruits and juices, justify further research. Additional scientific investigations will provide a better understanding of the biological effects of dried fruits on major chronic diseases and their biological mechanisms of action will be useful for future dietary guidance for dried fruits.

## Figures and Tables

**Table 2:** Clinical trials reporting cardiometabolic effects of routine (≥4 weeks) dried fruit consumption.

References	Study Design	Duration (Week)	Participants (*n*)	Fruit (Dose)	Comparator	Findings
Sullivan et al. [45]	Crossover	4	Men and women with BMI 25–36 kg/m^2^ and ≥1 additional cardiometabolic risk factor, *n* = 55	Equal parts (~28 g each) dried plums, Mission figs, Deglet Noor dates, and raisins totalling ¾ cups/day	Energy-matched processed snacks (animal crackers and fruit snack gummies)	Dried fruits increased LDL-C (0.10 mmol/L) and non-HDL-C (0.12 mmol/L) and reduced HDL-C (−0.05 mmol/L) compared to baseline. Dried fruits increased fasting glucose compared to control (0.08 mmol/L). No between-group or within-group differences in total cholesterol, TAG, blood pressure, or insulin.
Tinker et al. [53]	Crossover	4	Men with elevated total cholesterol (5.2–7.5 mmol/L), *n* = 41	Dried plums, ~100 g/day (12 plums)	360 mL grape juice	Dried plums reduced LDL-C compared to grape juice (−0.17 mmol/L). No difference in total cholesterol, HDL-C, or TAG.
Clayton et al. [54]	Parallel	8	Men and women with BMI ≥ 25 kg/m^2^, *n* = 45	Dried plums, ~84 g/day	Energy-matched portion (200 kcal) of low-fat muffins	Dried plums reduced LDL-C compared to low-fat muffins (−24.5 mg/dL). Dried plums increased C-peptide compared to baseline (+1.56 ng/mL). No between-group or within-group differences in total cholesterol, HDL-C, blood pressure, TAG, insulin, or glucose.
Alalwan et al. [55]	Parallel	16	Men and women with T2D, *n* = 96	Dates (Khudary cultivar, tamar stage), 3 dates/day	Usual diet	Dates reduced total cholesterol compared to baseline (−0.209 mmol/L). No between-group or within-group differences in HbA1c, TAG, HDL-C, or LDL-C.
Shishehbor et al. [56]	Parallel	5	Men and women with elevated total cholesterol (>200 mg/dL) or TAG (>200 mg/dL), *n* = 38	Raisins, 90 g/day	Usual diet	Raisins reduced DBP compared to control group (−1.56 mm Hg). Raisins reduced LDL-C (−0.68 mmol/L) and total cholesterol (−0.72 mmol/L) compared to baseline. No between-group or within-group differences in SBP, HDL-C, or TAG.
Kanellos et al. [57]	Parallel	24	Men and postmenopausal women with T2D, *n* = 48	Corinthian raisins, 36 g/day	Usual diet	Raisins reduced DBP compared to the control group (−6 mm Hg). No between-group or within-group differences in SBP, total cholesterol, LDL-C, HDL-C, TAG, fasting glucose, or HbA1c.
Anderson et al. [58]	Parallel	12	Men and women with BMI 25–34.9 kg/m^2^, blood pressure > 120/80 mm Hg, and fasting glucose 90–150 mg/dL, *n* = 46	Raisins, 3 ounces/day	Energy-matched pre-packaged processed snacks (three 100 kcal packages)	Raisins reduced SBP (−5.4 mmHg vs. baseline; −6.3 mmHg vs. snacks), DBP (−5.5 mmHg vs. baseline; −3.6 mmHg vs. snacks), HDL-C (−3.6 mg/dL vs. baseline), and HbA1c (−0.12% vs. baseline; −0.08% vs. snacks). No between-group differences in total cholesterol, LDL-C, TAG, or fasting glucose.
Bays et al. [59]	Parallel	12	Men and women with T2D and BMI 25–50 kg/m^2^, *n* = 46	Raisins, 3 ounces/day	Energy-matched pre-packaged processed snacks (three 100 kcal packages)	Raisins reduced SBP compared to snacks (−8.7 mm Hg). No between-group differences in fasting glucose, HbA1c, DBP, total cholesterol, LDL-C, HDL-C, or TAG.
Peterson et al. [60]	Crossover	5 (per arm)	Men and women with LDL-C 100–189 mg/dL and BMI 18.5–35 kg/m^2^, *n* = 102	Dried California Mission figs (~120 g/day, 12–15 figs)	Usual diet	Figs increased total cholesterol compared to control (6 mg/dL). No difference in LDL-C, HDL-C, or TAG.

Abbreviations: BMI, body mass index; DBP, diastolic blood pressure; HbA1c, hemoglobin A1c; HDL-C, high-density lipoprotein cholesterol; LDL-C, low-density lipoprotein cholesterol; SBP, systolic blood pressure; T2D, type 2 diabetes; TAG, triacylglycerols.

## Data Availability

No new data were created in this study. Data sharing is not applicable to this article.
